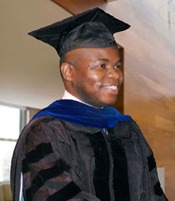# Obituary: in memoriam, Dr Atashili Julius (1977-2015)

**DOI:** 10.11604/pamj.2016.23.13.8794

**Published:** 2016-01-22

**Authors:** The Pan-African Medical Journal

**Affiliations:** 1 Pan-African Medical Journal, Yaoundé, Cameroon

**Keywords:** Atashili Julius, Cameroon, medical epidemiologist

## Abstract

The Pan-African Medical Journal (PAMJ) regrets to announce the death of Dr. Atashili Julius on 24 October 2015 in Douala, Cameroon, at the age of 38 years. He joined the PAMJ editorial board since 2009 and was one of his most distinguished scholars.

## Obituary

The Pan-African Medical Journal (PAMJ) regrets to announce the death of Dr. Atashili Julius on 24 October 2015 in Douala, Cameroon, at the age of 38 years. He joined the PAMJ editorial board since 2009 and was one of his most distinguished scholars.

Julius was absolutely one of a kind: humble, gentle, loving, respectful, hardworking, disciplined, and very intelligent. The perfect example to emulate. Most people who knew Dr Julius Atashili would agree with this statement from his first cousin Doris Atashili. A native of Cameroon, Dr. Atashili graduated from the University of Yaoundé-I with a Doctorate in Medicine in 2002. He then earned a Master in Public Health (2005) and a PhD in Epidemiology (2009) from the University of North Carolina at Chapel Hill, USA. He was passionate about improving population's health though research and training of medical personnel.

Speaking in Chapel Hill in 2009 about his work on cervical cancer among HIV-infected women, Dr. Atashili said "there needs to be more effort in the developing world to reduce morbidity and mortality from cervical cancer and that means improving diagnosis, training health providers in the diagnosis of precancerous lesions, and training lab cytologists. This will be the general direction of where I'm going". In addition to having a plan, Julius knew exactly how to go about it. In 2013, writing a memorial for his mentor, the late Professor Peter Ndumbe, he asserted in unequivocal terms that "the health of the populations can only be improved through research and publishing is a way of making available the evidence that would be used to improve health".

Although cut short prematurely, Dr. Atashili's had a very successful academic career. He joined the faculty of the University of Buea in 2009 and was instrumental in the development of the Department of Public Health. He authored or co-authored more than 40 manuscripts in peer-reviewed journals. Notably, he joined the PAMJ editorial team early in 2009 and played a key role in rapid expansion of this journal as author, reviewer and by promoting a culture of knowledge sharing and documentation among his peers and students.

Dr. Atashili was dedicated to the formation of the next generation of public health professionals, and he was sometimes mentoring 20 master students in a year. "Julius was an excellent teacher, a dedicated supervisor, a committed leader and mentor" said Dr. Agbornkwai Nyenty, one of his former students. "He saw potential in each and every one of his student, and was devoted to support their work responding to emails even at 3 am" said Mr. Ettamba Agborndip, another former student. According to Dr Eloumou of the Douala General Hospital (Cameroon), a former colleague and a friend "Julius was also very generous and selfless; every time I asked him for help with analysis for my manuscripts or anything else, he was always available and never asked for anything in return".

The scientific community and the PAMJ family lost a star. The remarkable Dr. Atashili Julius will be profoundly missed, by his family, by his students, by his former classmates, by his colleagues, and by the scientific community both in Africa and globally.